# Antimicrobial resistance in Germany and Europe – A systematic review on the increasing threat accelerated by climate change

**DOI:** 10.25646/11404

**Published:** 2023-06-01

**Authors:** Annika Meinen, Sara Tomczyk, Flora Noelle Wiegand, Muna Abu Sin, Tim Eckmanns, Sebastian Haller

**Affiliations:** Robert Koch Institute, Berlin, Germany Department of Infectious Disease Epidemiology

**Keywords:** CLIMATE CHANGE, ANTIMICROBIAL RESISTANCE AND CONSUMPTION, HEALTHCARE-ASSOCIATED INFECTIONS

## Abstract

**Background:**

Antimicrobial Resistance (AMR) is one of the top ten global public health threats facing humanity, alongside climate change. Here, we aim to summarise the effects of climate change (i.e. raise of temperature, change in humidity or precipitation) on spread of antibiotic resistance and on infections with antibiotic-resistant bacteria in Germany.

**Methods:**

We conducted a literature search with articles published between January 2012 and July 2022. Two authors screened titles, abstracts and full texts and extracted the data systematically.

**Results:**

From originally 2,389 titles, we identified six studies, which met our inclusion criteria. These studies show that an increase in temperature may lead to higher antibiotic resistance rates and an increased risk of colonisation as well as spread of pathogens. Furthermore, the number of healthcare-associated infections increases with increased temperature. Data indicate that higher antibiotic use is present in areas with warmer mean temperature.

**Conclusions:**

European data are scarce, but all studies identified point towards an increasing AMR burden due to climate change. However, further studies are needed to draw attention to the links between climatic factors and AMR and develop targeted preventive measures.

## 1. Introduction

Climate change has a clear impact on disease burden globally, and there is sufficient evidence to act now to mitigate the climate crisis. However, it remains challenging to quantify the impact of climate change on health for many diseases. The complexity of climate-dependent modifying factors of infectious diseases challenges the precision of modelling pathways and subsequent impacts.

Since the availability of antimicrobials, infections due to bacteria, viruses, fungi and parasites have become treatable. However, due to the misuse and overuse of antimicrobials, these pathogens become increasingly resistant. In 2019, the World Health Organization (WHO) declared antimicrobial resistance (AMR) one of the top ten global public health threats facing humanity, alongside climate change [1, 2]. According to a recent study on the global burden of AMR, an estimated 4.95 million deaths were associated with bacterial AMR (i. e. deaths due to infection with antimicrobial-resistant bacteria for which AMR may/may not be the cause) in 2019. Attributable deaths to bacterial AMR (i. e. deaths due to untreatable AMR infection, caused by drug resistance) accounted for 1.27 million deaths [3]. In European countries, Cassini et al. [4] estimated the burden of infections with antibiotic-resistant bacteria in 2015 and identified four antibiotic-resistant bacteria with the largest effect on human health: third-generation cephalosporin-resistant *Escherichia coli* (*E. coli*), meticillin-resistant *Staphylococcus aureus* (*S. aureus*), carbapenem-resistant *Pseudomonas aeruginosa* (*P. aeruginosa*) and third-generation cephalosporin-resistant *Klebsiella pneumoniae* (*K. pneumoniae*). These findings were recently confirmed by Mestrovic et al. [5] in another European systematic analysis in 2019, which highlighted regional differences in AMR burden, showing that the burden of AMR is higher in countries in the Mediterranean region, like Greece or Italy, compared to countries in the Northern European region, which could be linked to differences in climate among other factors [4]. Furthermore, the Organisation for Economic Co-operation and Development (OECD) estimated that 75% of the AMR burden derives from healthcare-associated infections (HAIs) [6].

Temperature, which may increase due to climate change [7], is known to have an effect on bacterial growth and reproduction; the optimal growth temperature for many key bacteria is above 30°C [8]. There is some evidence that plasmid transfer and potentially gene transfer of resistance genes are facilitated by increased temperature [9]. Hints for the relationship between climatic factors such as temperature and AMR have also been recently found. For example, MacFadden et al. [10] found that AMR in common pathogens such as *S. aureus*, *E. coli* and *K. pneumoniae* increased with increasing temperature.

In this review, we aim to summarise the key effects that climate change may have on the spread and burden of AMR among humans in Germany and Europe. We conducted a literature review and adapted a graphical model to support this assessment.

## 2. Methods

We conducted a literature search limited to peer reviewed articles published between January 2012 and July 2022 in English, French or German. PubMed was searched using the keywords climate change, temperature, healthcare-associated infection (HAI), and antimicrobial resistance, including the MeSH terms for climate change, global warming, climatic processes, antibacterial agents, and drug resistance (bacterial). The complete search string is documented in the Annex. Further literature was retrieved from citation screening of selected articles. Studies were restricted to those including countries of the European Union (EU) and/or European Economic Area (EEA) and to primary analyses or reviews (e. g. editorials were excluded) for this review. This review considers the Preferred Reporting Items for Systematic Reviews and Meta-Analyses (PRISMA) Statement [11].

Two authors (AM and FW) independently of each other screened titles, abstracts and full texts and extracted the following data systematically: citation, study period, study design, demographics, climate indicators, definition of outcome as pathogen and/or antimicrobial resistance and/or infections analysed. Discrepancies between the reviewers were solved by discussion until a consensus was reached. Data were summarised in tables and stratified by drug resistance, pathogen and type of infection.

Additionally, we utilised the webtool provided by Mora et al. [12] to further illustrate the impact of climate change on AMR pathogens. Based on a comprehensive global review of available literature and collected data, the tool allows the user to model pathways in which climatic hazards, through different transmission routes, impact specific diseases. The authors comprehensively assessed the evidence from climatic hazards worldwide. We adapted the model using this tool by concentrating on the main AMR pathogens responsible for the European AMR burden highlighted by Cassini et al. [4].

## 3. Results

**Figure 1:**
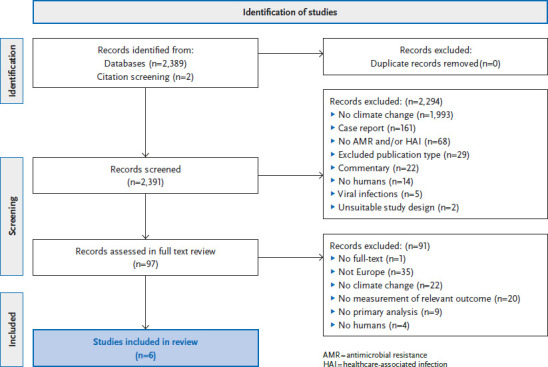
Depiction of literature selection adapted from the PRISMA Flow Diagram [11]

Our search identified a total of 2,389 titles (Figure 1). After eliminating duplicates and screening the remaining titles and abstracts, 97 publications were left for full text evaluation. Of these, only six studies met our selection criteria (Table 1). Due to our focus on the situation in Germany and EU/EEA countries, we excluded the non-European studies for this summary (n=35). Most eligible studies were observational studies.

### 3.1 Body of evidence for association of increases in temperature and occurrence of AMR

Using data from the European Antimicrobial Resistance Surveillance Network (EARS-Net), McGough et al. [14] showed that AMR in *E. coli* and *K. pneumoniae* increased from 2000 to 2016, whereas meticillin-resistant *S. aureus* (MRSA) decreased over time. The reduction of MRSA detection is commonly attributed to targeted public action and, thus, may not be explained by climate indicators [19]. Kaba et al. [13] and McGough et al. [14] show a correlation between warmer temperature (whether expressed as mean temperature or as minimum ambient temperature) and an increase in antibiotic resistance. Warm season mean temperature was identified as a predictor for MRSA, multi-drugresistant *E. coli* (MREC) and carbapenem-resistant *K. pneumoniae* (CRKP). With increases in mean temperature, the rates of MRSA, MREC and CRKP were shown to increase [13]. Furthermore, McGough et al. [14] demonstrated that AMR increases faster at higher temperatures. In warmer countries, where mean temperature is 10°C higher than the overall mean temperature in European countries, an increased rate of change of AMR by 0.33% per year for aminoglycoside-resistant *E. coli* and 0.55% per year for third-generation cephalosporin-resistant *E. coli* was observed*.* For fluoroquinolone-resistant *E. coli*, an increase of 0.57% per year was found after accounting for other recognised resistance drivers including antibiotic consumption and population density*.* For *K. pneumoniae*, even higher increases were identified: 0.9% per year for third-generation cephalosporin-resistant *K. pneumoniae* and 1.2% for fluoroquinolone-resistant *K. pneumoniae*. McGough et al. [14] concluded that ambient temperature might considerably influence antibiotic resistance growth rates. In a review, Forrester et al. [15] also concluded that temperature increase due to climate change causes an increase in resistance in pathogenic bacteria.

### 3.2 Body of evidence for association of increases in temperature and humidity with bacterial pathogen growth and spread

The optimal growth temperature for many relevant bacteria is above 30°C [8], so increased bacterial proliferation with increasing temperatures seems likely. In their review, Forrester et al. [15] state that higher temperatures and humidity increase colonisation and infection risk of MRSA. Seasonality was also reported to affect colonisation or infection with *Clostridioides difficile*, varying by the Southern and Northern hemisphere. Although they found no data supporting associations of temperature, humidity or seasonality with carbapenem-resistant Enterobacteriaceae (CRE), they hypothesised that such associations could also be plausible [15]. Surveillance studies by Aghdassi et al. [16, 18] and Schwab et al. [17] suggested that changes in the microbiome composition could potentially be modified by rising temperatures. Thus, bacterial pathogen growth and spread may be facilitated by increased temperatures, even though the mechanisms underlying the hypothesis that to a certain extent higher temperatures lead to more bacterial proliferation are not fully understood.

### 3.3 Body of evidence for association of increases in temperature and humidity with healthcare-associated infections

There is also evidence that the number of HAIs increases with increased temperature. This is of relevance because 75% of the AMR burden stem from HAIs, which result in higher use of antibiotics [6]. Surgical site infections (SSIs) belong to the most common HAIs with an estimated 800,000 cases per year in the EU. Most frequently found pathogens in SSIs are *S. aureus*, *Enterococcus* spp. and *E. coli* [18]. In particular, Aghdassi et al. [16] showed that SSIs occur more often after surgeries in warmer months (≥20°C) compared to months with colder temperatures (<5°C) (adjusted Odds Ratio (AOR): 1.13, 95% confidence interval (Cl): 1.06–1.20). SSIs increased when temperatures rose above 20°C during the month of surgery with both gram-negative and gram-positive pathogens. When considering temperature as a continuous variable, data showed that the likelihood of SSI occurrence increases by 1% per 1°C increase in temperature. Stronger associations between warmer temperatures and an increase in SSIs can be found in gram-negative pathogens. Furthermore, superficial SSIs appeared to have a higher temperaturerelated association than deeper SSIs. Superficial SSIs with gram-negative pathogens occurred 38% more frequently while temperatures were ≥20°C, compared to months with temperatures <5°C [16]. In a second study, Aghdassi et al. [18] found an increased risk of 6% for SSI with *Acinetobacter baumannii* per 1°C increase and a 4% risk for SSI with *Enterobacter* spp. in Germany. There was no association found between risk of SSI with *Streptococcus* spp. or *Candida albicans* and an increase in temperature.

Another study by Schwab et al. [17] reported on healthcare-associated primary bloodstream infections (ha-BSIs) in intensive care units (ICUs) and identified an association between the increase in mean daily temperature and ha-BSIs. In months with temperatures ≥20°C, the incidence rate of ha-BSIs was 17% higher than in months with temperatures <5°C. The effect of temperature is most prominent for ha-BSIs with gram-negative pathogens (38% incidence rate increase), followed by ha-BSIs with gram-positive pathogens (13% incidence rate increase). The only exception was found in *Streptococcus pneumoniae* with a reduced incidence rate ratio (IRR) of 0.498 (95% Cl: 0.174–1.429) for temperatures ≥20°C compared to temperatures <5°C. This may well be explained by its transmission pathway via droplets, which is more relevant when the population stays predominantly indoors during colder seasons. In addition to temperature, relative humidity inversely correlated with an increase in ha-BSIs [17].

### 3.4 Body of evidence for association of increases in temperature with antibiotic consumption

In the European surveillance study, McGough et al. [14] presented data indicating that higher antibiotic use is present in countries with higher mean temperatures. In Germany, Aghdassi et al. [18] suggested that an increase in HAIs may be expected when temperatures rise and these rising infection rates would then potentially lead to increased use of antibiotics.

### 3.5 Summary of climatic hazards that could aggravate AMR burden in Europe

**Figure 2:**
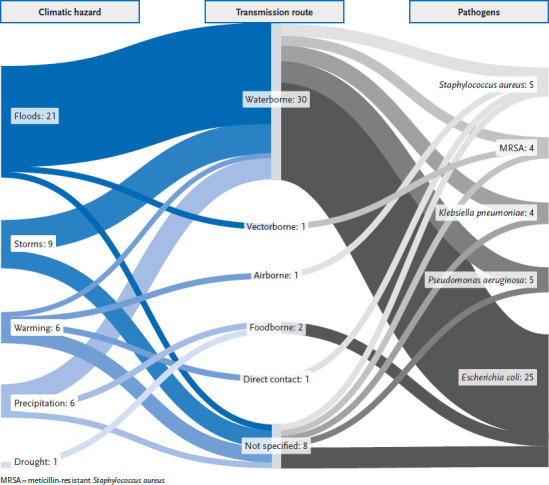
Summary of climatic hazards that may modify the spread and health burden of resistant pathogens. Full interactive display of all pathways and the underlying data are available [20]. Source: Webtool by Mora et al. [12, 20]

In a recent study on the impact of climate hazards on pathogenic human diseases, Mora et al. [12] collected data on pathways in which climatic hazards, through different transmission routes, result in aggravation of specific diseases. They identified 43 articles worldwide with respect to climate change and information on pathogens causing the highest AMR burden in Europe according to Cassini et al. [4]. The results of these studies can be illustrated in a Sankey diagram of potential pathways through which climatic hazards could aggravate these pathogens (Figure 2). The most common pathogens found with an association to climatic hazards included *E. coli* (with 25 publications), followed by *S. aureus* (nine publications, four publications specifically for MRSA), *P. aeruginosa* (five publications) and *K. pneumoniae* (four publications). For these pathogens, the associated climatic hazards were floods, storms, warming, precipitation, and droughts. The main routes of transmission were waterborne transmission as well as unspecified transmission.

## 4. Discussion

### 4.1 Climate change and AMR

Climate change, through increases in temperature and changes in humidity and precipitation, will likely lead to bacterial pathogen spread, increased use of antibiotics and increased AMR in Europe. However, due to the complexity of interactions and the significance of various factors, we were not able not elucidate the full dimension of these associations. European data are scarce, and we were only able to identify six studies that met our inclusion criteria. However, all six of these studies pointed towards an overall further acceleration of increasing AMR burden due to climate change.

There is some evidence specifically demonstrating the increase in AMR with increasing temperatures in Europe [13, 14]. Studies outside of Europe verify these results. A study from the United States by MacFadden et al. [10] found that an increase in local minimum temperature is associated with increasing antibiotic resistance rates. However, this does not apply to all pathogens, and further factors like hygiene measures need to be considered. EARS-Net data reveal decreasing MRSA incidence in Europe, for example [14]. Hansen et al. [21] found that countries with decreasing MRSA proportions showed especially strict implementation of various prevention measures. The studies considered in our review also observed an increase in HAIs associated with an increase in temperature. SSIs and ha-BSIs in Germany, mostly due to gram-negative bacteria, occurred more frequently in warmer temperatures (≥20°C). In line with these findings, a study from Japan by Kobayashi et al. [22] concluded that SSIs are associated with summer months (July–September, Hazard Ratio: 1.53, 95% CI: 1.06–3.83). Moreover, SSI rates were higher in summer months compared to non-summer months (3.9% vs. 1.9%, p<0.05). However, another study from Japan found contrasting results: Sagara et al. [23] ascertained that infections of the eye were most frequent in the winter months and less frequent in the summer and autumn months. Yet, when they stratified results by pathogens, they found that infections caused by *S. aureus* were more frequent in summer and autumn compared to colder seasons. *Streptococcus* spp. infections were more common in spring and summer months. Another surveillance paper concentrating on AMR genes and their distribution on a global level, taking climatic factors into account, found an uneven distribution of AMR genes in bacteria across several cities worldwide, suggesting an association with higher temperatures in different regions [24]. More evidence is needed to fully understand the associations between climatic factors and AMR, but existing evidence is sufficient to foresee that without appropriate interventions, climate change and the burden of AMR will continue to increase.

Climate hazards can bring humans and disease-causing organisms closer together, leading to a rise in pathogen transmission and infections, as demonstrated in our illustrated model with data from Mora et al. [12] (Figure 2). An increase in extreme weather events and natural disasters may cause disruptions and conditions that lead to an increase of AMR and pathogen spread as well as an increase in related antibiotic use. Such events can also lead to population displacement and increased burden on health systems which can further aggravate the spread of AMR and HAIs [25]. We focused on the AMR bacterial pathogens currently causing the highest burden in Europe. However, beyond the burden of infections with antibiotic-resistant bacteria, resistance to antimicrobial drugs such as resistance to antimalarial medications or antiretroviral therapy may also be increased by climate change [26]. Furthermore, the climate crisis could allow for the emergence and spread of new and re-emerging threats. For example, it has been suggested that *Candida auris*, a fungal pathogen that is often multi-drug-resistant and exists in the environment, may become increasingly pathogenic due to climate change [27]. In addition, recent studies indicate a potential threat for public health caused by the release of bacteria as well as viruses from thawing permafrost due to defrosting as a result of climate change [28, 29].

### 4.2 One Health and AMR

**Figure 3:**
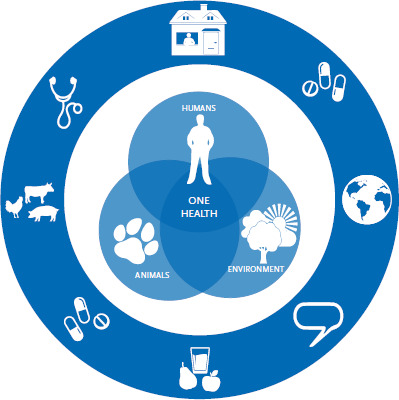
One Health drivers and interactions related to infectious diseases and antimicrobial resistance. Humans, animals and the environment (centre) influence AMR through drug use in humans and animals, nutrition, livestock, communication, healthcare personnel and facilities (outer circle). Illustration: Robert Koch Institute

Human pathogen colonisation and infection can result from exposure to pathogens across a range of community and healthcare system domains and may be influenced by socioeconomic and environmental determinants of health. With our focus on human health, we did not comprehensively assess the changes in AMR from a One Health perspective, although we partly highlight this aspect by taking the review by Forrester et al. [15] into account, which includes the One Health perspective. AMR is rising in humans, animals, plants and the environment [30], and these increases may all be influenced by global and local temperatures rising due to climate change. One Health drivers (i. e. human and animal populations as well as the environment) and interactions related to infectious diseases and AMR can be illustrated as seen in Figure 3. Such interactions can often only be retrospectively analysed and their significance is likely to be underestimated in studies. As current evidence is pointing towards the aggravation of the existing AMR pandemic due to climate change, better data and public health action in human, animal, and environmental health domains are needed.

### 4.3 Limitations of this review

Possible limitations of our analysis are related to the limited number of studies included in this review. Among the excluded 35 non-European studies, there may be additional relevant evidence to better understand the association of climate change and AMR.

For included studies, the predominant study type was observational, where surveillance data of pathogens or infection were combined with climate data in form of an ecological study design. Here, measuring errors may occur in exposure and outcome leading to risk of bias and imprecision of the reported estimates. Even though various confounders were addressed within the single studies, there may be additional factors with influence on the association of climate and AMR or HAIs. By focusing on EU/EEA countries in this review, confounding due to societal and economic factors may have been reduced. Nonetheless, their potential relevance for AMR has been demonstrated before and should be kept in mind.

The two publications by Aghdassi et al. [16, 18] included in our review, should be considered together, as they use the same dataset. Those studies, as well as the one by Schwab et al. [17] were included, as we assume that an increase of HAIs will consecutively lead to an increasing AMR burden and thus may be considered as indirect evidence.

## 5. Conclusion and recommendations

The links between AMR and climate change require significantly more attention. Further detailed studies with local data on weather conditions, resistance situation, infectious diseases, but also antibiotic use, social determinants and other infectious and social factors are needed to better understand the pathways and subsequent impacts of these associations. Financial and scientific resources need to be made available to realise these studies. The example of decreasing MRSA incidence in many EU countries due to targeted public health action, in particular infection prevention and control measures in hospitals, is a reassuring sign that the AMR pandemic can be controlled. But climate change will likely make the control of AMR more challenging. On the other hand, there may be synergistic effects (or co-benefits) when leveraging action against both AMR and climate change, for example through reduction of industrial livestock farming or meat production and consumption. Although this summary focused on evidence in Europe, AMR is a pandemic threat. The Global South often suffers from a higher burden of AMR. Thus, prevention and control of AMR will have limited success with single country interventions and needs a global focus.

Despite the complexity of climate change processes and AMR, it will be important to closely monitor the changes in AMR over time in order to inform the prioritisation of public health measures. Intensified One Health surveillance approaches will be needed. Global surveillance systems such as the European EARS-Net and the worldwide Global Antimicrobial Resistance and Use Surveillance System (GLASS) are particularly important for country comparisons and could enable ecological studies analysing associations with regional climate change. Globally, we will have to further improve the availability and standardisation of antimicrobial susceptibility testing (AST), particularly in the Global South, to allow for more representative AMR surveillance data. In addition, AMR burden of disease studies will have to be developed and routinely updated to inform timely public health decision-making.

In addition to improved One Health AMR surveillance, universal health coverage and effective infection prevention and control measures including reliable access to water, sanitation and hygiene and One Health antimicrobial stewardship are needed to control the AMR pandemic worldwide. Investment in research and development on new antimicrobial drugs is needed as well as on the development of relevant vaccines.

## Key statement

- Climate change and AMR are two of the biggest and most complex threats facing the world. Both have been exacerbated by, but can be contained through, human action.
- Increases in temperature and changes in humidity and precipitation may increase the spread of AMR and healthcare-associated infections.
- Climate change contributing to more frequent and severe extreme weather events will likely result in increased antimicrobial drug use in humans and animals.
- Overuse of antimicrobial drugs across human, animal and environmental sectors exacerbates AMR.
- More financing, political advocacy and coordinated global action are needed to better respond to the threats of antimicrobial resistance and the climate crisis.

## Figures and Tables

**Table 1:** Information on the six studies that met inclusion criteria of this review. Studies are sorted by author, measured exposure (climate factors), outcome (AMR and/or infection), and results are displayed.

First author and publication year	Observational period	Country	Data source	Study design	Exposure measurement	Outcome measurement	Results
**Kaba** [13], **2020**	2011 – 2016	30 countries (all EU and EEA members, in addition to Iceland and Norway)	EARS-Net Surveillance data (routine AST results are collected from clinical laboratories by the national network); historical temperature data	Observational ecological study	Historical monthly mean temperature	Annual national AMR prevalence (CRPA, CRKP, MREC, MRSA)	Significant correlation between warm season (May–October) mean temperature and MRSA (Rs=0.826), MREC (Rs=0.718) and CRKP (Rs=0.798); correlation between cold season (November–April) mean temperature and MRSA (Rs=0.691); correlation with warm season net change in temperature and CRPA (Rs=0.748) and MREC (Rs=0.617); **warm season mean temperature is a significant predictor of MRSA, MREC and CRKP, but not CRPA**
**McGough** [14], **2020**	2000 – 2016	28 European countries (all EU and EEA members in addition to Iceland, Norway and United Kingdom)	EARS-Net Surveillance data (routine AST results are collected from clinical laboratories by the national network); modelled and assimilated meteorological data, available at Modern-Era Retrospective analysis for Research and Applications, Version 2 (MERRA-2)	Observational ecological study	Annual minimum ambient temperature	Annual national AMR prevalence (aminopenicillins (*E. coli*), third-generation cephalosporins (*E. coli and K. pneumoniae*), fluoroquinolones (*E. coli and K. pneumoniae*), aminoglycosides (*E. coli and K. pneumoniae*), and meticillin (*S. aureus*))	AMR in *E. coli* & *K. pneumoniae* increased over time for most European countries, MRSA generally decreased over time; positive linear association between minimum ambient temperature and AMR across all countries, years, pathogens, and antibiotic subclasses; **relationship between temperature and resistance increases with time and AMR increases faster at higher temperatures**
**Forrester** [15], **2022**	1990 – 2020	22 countries: 15 European countries, 7 low- and middle income countries	101 publications	Review	Temperature, humidity, seasons	Infection or colonisation with common antibiotic-resistant or antibiotic-associated pathogens (MRSA, *C. difficile*, CRE)	MRSA: **higher temperatures and humidity** have been documented to increase colonisation and infection **with MRSA**; *C. difficile*: **Seasonality** has been reported to **affect colonisation or infection with *C. difficile*** and varies by Southern (October–November) and Northern Hemisphere (March–April)
**Aghdassi** [16]*, **2019**	2000 – 2016	Germany	Surgical procedures and SSI from KISS; meteorological monitoring station data of DWD	Observational ecological study	Monthly mean temperature	Rates of SSIs per 1,000 surgeries	Number of SSIs per 1,000 surgeries increased with higher temperatures; **SSI at temperatures ≥20°C more likely compared to temperatures <5°C (AOR: 1.13 [95% Cl: 1.06–1.20])**; occurrence of superficial SSI with gram-negative pathogens up to 38% more likely with temperatures ≥20°C (AOR: 1.38 [95% Cl: 1.16–1.64]) compared to temperatures <5°C; number of SSIs per 1,000 surgeries increased by 1% per 1°C increase
**Schwab** [17], **2020**	2001 – 2015	Germany	SSIs from ICUs participating in ‘ICU-KISS’ module of KISS; climate station network data of DWD	Observational ecological study	Daily mean temperature, daily maximum temperature, daily precipitation, relative humidity, and the daily duration of sunshine	Incidence of primary healthcare-associated bloodstream infections (ha-BSIs) stratified by pathogens per 10,000 patient days	**Incidence of ha-BSIs 17% (IRR 1.169 [95% Cl: 1.077-1.269]) higher in months >20°C compared to months <5°C**; this effect is one third (38%) higher for gram-negative pathogens and 13% higher for gram-positive pathogens; *S. pneumoniae* occurred 50% less frequently at months >20°C than at <5°C.
**Aghdassi** [18]*, **2021**	2000 – 2016	Germany	Surgical procedures and SSI from ‘OP-KISS’ module of KISS, data from DWD	Observational ecological study	Monthly mean temperature	Rates of surgical site infections (SSIs) per 1,000 operations stratified by pathogens (*S. aureus*, *Enterococcus* spp., coagulase-negative staphylococci, *Streptococcus* spp., *Corynebacterium* spp., *E. coli*, *P. aeruginosa*, *Enterobacter* spp., *Klebsiella* spp., *Proteus* spp., *Bacteroides* spp., *Citrobacter* spp., other Enterobacteriaceae, *Serratia* spp., *Acinetobacter* spp., *C. albicans*)	**Correlation between higher temperatures and occurrence of SSIs; increase in SSI rate per additional 1°C for almost all pathogens excluding *Streptococcus* spp. and *C. albicans*;** strongest association for risk for SSIs with *Acinetobacter* spp. (6% increase per additional 1°C) and *Enterobacter* spp. (4% increase per additional 1°C); risk for SSIs caused by *Acinetobacter* spp. and *Enterobacter* spp. increased >2-fold in months with ≥20°C compared to <5°C

AMR=antimicrobial resistance, AOR=adjusted odds ratio, AST=antimicrobial susceptibility testing, *C. albicans*=*Candida albicans*, *C. difficile*=*Clostridioides difficile* CI=confidence interval,

CRE=carbapenem-resistant Enterobacteriaceae, CRKP=carbapenem-resistant *K. pneumoniae*, CRPA=carbapenem-resistant *P.aeruginosa*, DWD=Deutscher Wetterdienst (German Meteorological Service),

EARS-Net=European Antimicrobial Resistance Surveillance Network, EEA=European Economic Area, EU=European Union, ha-BSI=healthcare-associated bloodstream infection, ICU=intensive care unit,

IRR=incidence rate ratio, KISS=Krankenhaus-Infektions-Surveillance-System (German Nosocomial Infection Surveillance System), MRSA=meticillin-resistant *S. aureus*, MREC=multi-drug-resistant *E. coli*,

Rs=Spearman rank correlation coefficient, SSI=surgical site infection, *S. pneumoniae*=*Streptococcus pneumoniae*

*Both articles used the same data set and should be considered together.

**Annex Table 1:** Applied search string

Database	Search strategy
PubMed	(“climate change”[MeSH Terms] OR “global warming”[MeSH terms] OR “climatic processes”[MeSH terms] OR (“climate*”[Title/Abstract] AND “change*”[Title/Abstract]) OR “climate*”[Title/Abstract] OR “temperature*”[Title/Abstract]) AND (“Anti-Bacterial Agents”[MeSH Terms] OR “Drug Resistance, Bacterial*” [MeSH Terms] OR “health care associated infection” [Title/Abstract] OR “nosocomial”[Title/Abstract] OR “AMR” [Title/Abstract] OR “antimicrobial resistance*”[Title/Abstract] OR “antimicrobial resistan*”[Title/Abstract] OR “antibiotic*”[Title/Abstract]) AND ((y_10[Filter]) AND (humans[Filter]))
